# Comprehensive leaf size traits dataset for seven plant species from digitised herbarium specimen images covering more than two centuries

**DOI:** 10.3897/BDJ.9.e69806

**Published:** 2021-07-13

**Authors:** Vamsi Krishna Kommineni, Susanne Tautenhahn, Pramod Baddam, Jitendra Gaikwad, Barbara Wieczorek, Abdelaziz Triki, Jens Kattge

**Affiliations:** 1 Max Planck Institute for Biogeochemistry, Jena, Germany Max Planck Institute for Biogeochemistry Jena Germany; 2 Ernst-Abbe-Hochschule Jena, Jena, Germany Ernst-Abbe-Hochschule Jena Jena Germany; 3 Friedrich Schiller University Jena, Jena, Germany Friedrich Schiller University Jena Jena Germany; 4 German Centre for Integrative Biodiversity Research (iDiv) Halle-Jena-Leipzig, Leipzig, Germany German Centre for Integrative Biodiversity Research (iDiv) Halle-Jena-Leipzig Leipzig Germany; 5 University of Sfax, Sfax, Tunisia University of Sfax Sfax Tunisia

**Keywords:** morphological leaf traits, leaf size, leaf length, leaf width, digital herbarium specimen, TraitEx, iDigBio, GBIF, TRY trait database, *Salix bebbiana* Sarg., *Alnus incana* (L.) Moench, *Viola canina* L., *Salix glauca* L., *Chenopodium album* L., *Impatiens capensis* Meerb. and *Solanum dulcamara* L.

## Abstract

**Background:**

Morphological leaf traits are frequently used to quantify, understand and predict plant and vegetation functional diversity and ecology, including environmental and climate change responses. Although morphological leaf traits are easy to measure, their coverage for characterising variation within species and across temporal scales is limited. At the same time, there are about 3100 herbaria worldwide, containing approximately 390 million plant specimens dating from the 16th to 21st century, which can potentially be used to extract morphological leaf traits. Globally, plant specimens are rapidly being digitised and images are made openly available via various biodiversity data platforms, such as iDigBio and GBIF. Based on a pilot study to identify the availability and appropriateness of herbarium specimen images for comprehensive trait data extraction, we developed a spatio-temporal dataset on intraspecific trait variability containing 128,036 morphological leaf trait measurements for seven selected species.

**New information:**

After scrutinising the metadata of digitised herbarium specimen images available from iDigBio and GBIF (21.9 million and 31.6 million images for *Tracheophyta*; accessed date December 2020), we identified approximately 10 million images potentially appropriate for our study. From the 10 million images, we selected seven species (*Salix bebbiana* Sarg., *Alnus incana* (L.) Moench, *Viola canina* L., *Salix glauca* L., *Chenopodium album* L., *Impatiens capensis* Meerb. and *Solanum dulcamara* L.) , which have a simple leaf shape, are well represented in space and time and have high availability of specimens per species. We downloaded 17,383 images. Out of these, we discarded 5779 images due to quality issues. We used the remaining 11,604 images to measure the area, length, width and perimeter on 32,009 individual leaf blades using the semi-automated tool TraitEx. The resulting dataset contains 128,036 trait records.

We demonstrate its comparability to trait data measured in natural environments following standard protocols by comparing trait values from the TRY database. We conclude that the herbarium specimens provide valuable information on leaf sizes. The dataset created in our study, by extracting leaf traits from the digitised herbarium specimen images of seven selected species, is a promising opportunity to improve ecological knowledge about the adaptation of size-related leaf traits to environmental changes in space and time.

## Introduction

Plant traits - the morphological, anatomical, physiological, biochemical and phenological characteristics of plants measurable at the individual plant level (Violle et al. 2007) - are vital to quantify, understand and predict plant and vegetation functional diversity and ecology (Grime 1974, McGill et al. 2006). Leaf attributes are amongst the most important traits as they provide relevant information about plant and ecosystem function (Funk et al. 2017, Poorter and Bongers 2006). Leaf area (the one-sided projected area of a leaf) is key to understanding the leaf energy balance, which affects photosynthesis and respiration rates (Wright et al. 2017). Leaf area is amongst the commonly sampled quantitative plant attributes and has more than 200,000 records in the TRY plant trait database (Kattge et al. 2020). Nevertheless, coverage is still limited, especially for characterising variation within species across geographical space and longer time-scales (Kattge et al. 2020). The paucity of data and representative nature limits the scientific community's ability to understand and predict species and ecosystem responses to environmental and climate change (König et al. 2019, Tautenhahn et al. 2020).

Approximately 390 million plant specimens are stored in 3100 herbaria worldwide (Thiers 2019). These specimens provide good biogeographical and temporal coverage - dating back to the 16th century and offering a "window into the past" (Meineke et al. 2018, Lang et al. 2019). However, useful observations earlier than the year 1850 are very few in numbers (Groom 2015). Globally, many herbaria are undertaking digitisation campaigns and are making digitised specimen images openly accessible via various biodiversity data platforms, such as the Global Plants Database (2.6 million images), Natural History Museum Paris (8 million images) (Kirchhoff et al. 2018), iDigBio (35.2 million images) and most of them are published through the GBIF network (41 million images; accessed date December 2020) with considerable overlap. Due to the increasing availability of digitised herbarium specimens, efforts, such as extracting species' phenological and trait information using machine learning approaches from these images, have increased (Carranza-Rojas et al. 2017, Willis et al. 2008, Younis et al. 2018, Weaver et al. 2020, Younis et al. 2020).

To characterise the variation of leaf size within species across time and space, we need specimen images with consistent information about sampling location and date. This information allows characterising the environment in which the specimens had grown using external data, for example, from gridded climate or soil databases (Fick and Hijmans 2017, Hengl et al. 2017, Taylor et al. 2012). Georeference and sampling date are often available with the digital images of the herbarium specimens - 9.1 million out of 35.2 million images from iDigBio and 11.1 million out of 41 million images from GBIF provide georeference and sampling date. Given the significant number of herbarium specimens and the increasing numbers of digitised herbarium specimen images, including metadata information, we here evaluate the potential to use this information to overcome data limitations for size-related leaf traits in space and time.

First, we identified the relevant biodiversity data platforms and analysed their metadata for images of *Tracheophyta* species with suitable leaves and sufficient additional information, i.e. sampling date and georeference. We selected seven species that were well represented in space and time. We downloaded the pertinent images and tested the applicability of a semi-automated tool, TraitEx (Gaikwad et al. 2019), to extract the leaf size traits: length, width, perimeter and area of individual leaf blades. This article describes the workflow to identify, select and download appropriate herbarium specimen metadata and images and extract leaf traits using the TraitEx software. We provide a comprehensive dataset of leaf size traits for seven species as an outcome of this approach. Finally, we compare the extracted measurements with data from the global plant trait database TRY.

## Sampling methods

### Study extent

Apart from the biodiversity data platforms mentioned earlier, there are several other institutions, libraries and herbaria, such as the Utah Valley State College Digital Herbarium, Moscow university herbarium, vPlants A Virtual Herbarium of the Chicago Region, The Virtual Herbarium of The New York Botanical Garden, WTU Herbarium Image Collection, OSU Type Specimen Images and Original Descriptions, which store digital herbarium specimen images. We assessed the publicly available metadata from these resources and it revealed that iDigBio and GBIF harvest the data from several institutions, libraries and herbaria worldwide to make the data openly available to the scientific community and society through their respective data platforms. Therefore, we decided to focus on extracting metadata information for the digital herbarium specimen images from iDigBio and GBIF (21.9 million and 31.6 million images only for *Tracheophyta*, respectively; accessed date December 2020).

### Sampling description

**Figure 1.**
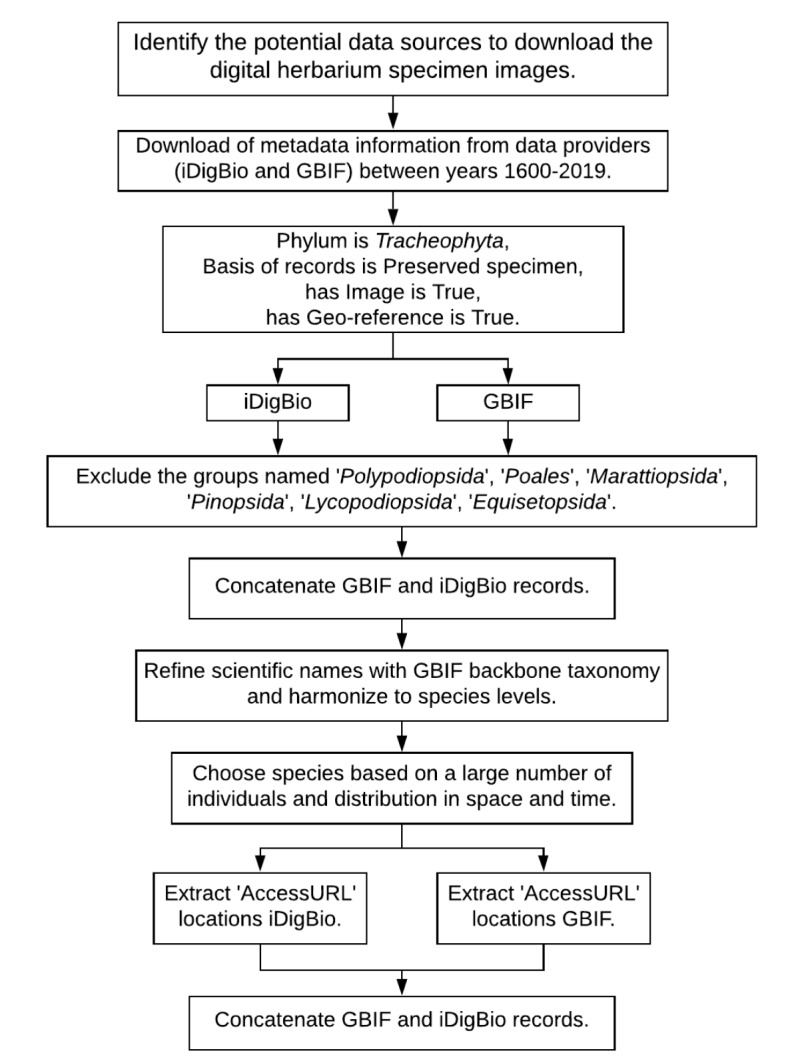
Flowchart for processing the metadata from iDigBio and GBIF on digitised herbarium specimens (Apart from the identification of data sources, all steps are automated using Python scripts).

**Figure 2.**
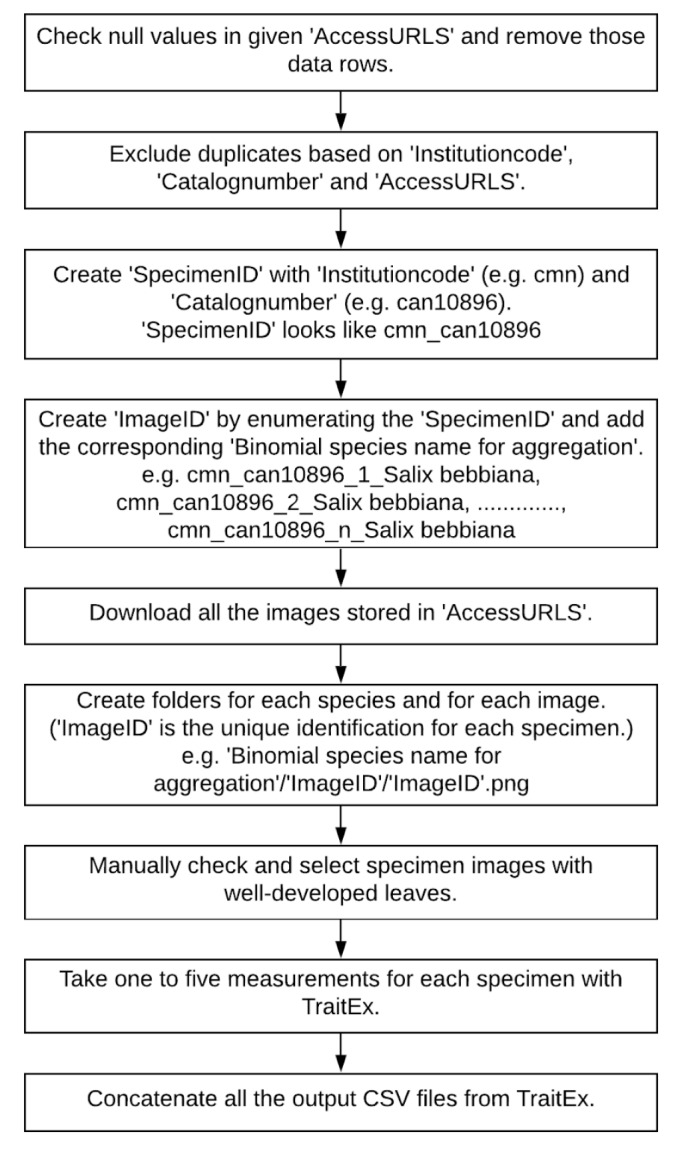
Workflow for downloading the images and measuring traits using TraitEx (Except for the leaf measuring process using TraitEx, all steps are automated using Python scripts).

Downloading a large number of available herbarium specimen images from the repositories takes a substantial amount of time. As a consequence, the images for trait extraction were selected in five steps (Figs 1, 2): (1) identification of specimens from GBIF and iDigBio with sufficient metadata information; (2) harmonising names to species level, based on the GBIF backbone taxonomy; (3) selecting appropriate species for our study; (4) acquisition of image URLs and exclusion of duplicates; (5) download of images and final selection for trait measurements.

The workflows for metadata extraction, downloading digital herbarium specimens and trait measurements using TraitEx are shown in Fig. 1 and Fig. 2.

**Identification of specimens from GBIF and iDigBio with sufficient metadata information**: We extracted the metadata for potentially applicable specimens from iDigBio and GBIF using the idigbio and pyGBIF libraries of the programming language Python for the years 1600 to 2019. For iDigBio, we used the following predefined search parameters (from idigbio API): ‘PreservedSpecimen’ for ‘basisofrecord’, ‘true’ for ‘has image’, ‘plantae’ for ‘kingdom’, {‘type’: ‘exists’} for ‘scientificname’ and {‘type’: ‘exists’} for ‘geopoint’. For GBIF, we used the following predefined search parameters (from pyGBIF API): 6 for ‘kingdomkey’ (kingdomkey 6 is Plantae), 7707728 for ‘phylumkey’ (phylumkey 7707728 is *Tracheophyta*), ‘StillImage’ for ‘mediatype’, True for ‘hasCoordinate’ and ‘PRESERVED_SPECIMEN’ for ‘basisOfRecord’. The following metadata: 'Source', 'Institutioncode', 'Catalognumber', 'UUID' and 'GBIFID' characterise specimen identity; 'Scientific Names', 'Family', 'Order', 'Class', 'Phylum' characterise specimen taxonomy; 'Latitude (from iDigBio and GBIF)', 'Longitude (from iDigBio and GBIF)' and 'Sampling date' characterise specimen georeference and sampling date. Missing metadata were replaced with the string ‘NA’ (Not Available). See section "Data resources" for a description of the metadata attributes. Records with missing information for latitude, longitude or sampling date were excluded.

Records from the taxonomic groups *Polypodiopsida*, *Poales*, *Marattiopsida*, *Pinopsida*, *Lycopodiopsida* and *Equisetopsida* were excluded after the download of metadata information because their leaf sizes or shapes were considered problematic for trait measurements.

**Figure 3.**
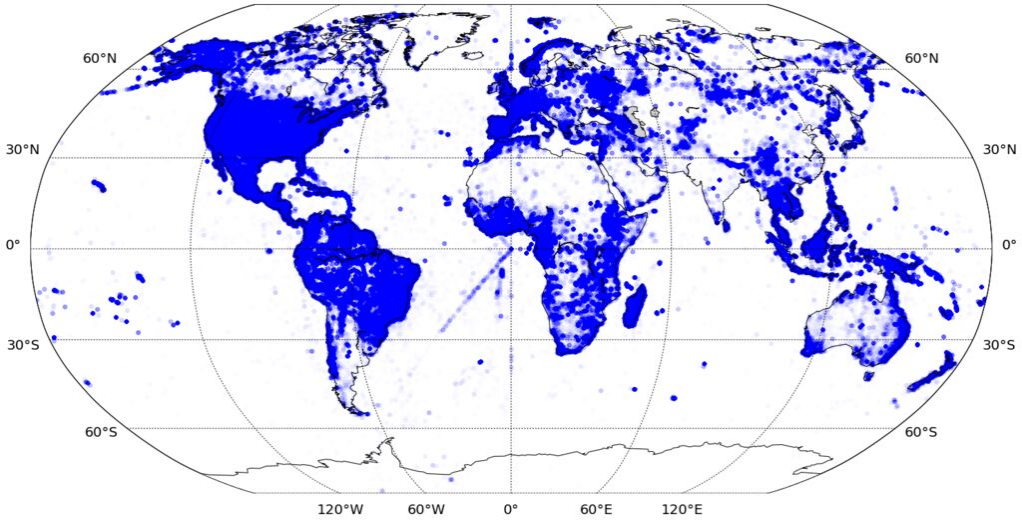
Spatial distribution of metadata for 9,998,299 digital herbarium specimen images from iDigBio and GBIF for *Tracheophyta* (excluding *Polypodiopsida, Poales, Marattiopsida, Pinopsida, Lycopodiopsida* and *Equisetopsida)* with georeference and sampling date available (for more details, refer to section 'Sampling methods').

**Figure 4.**
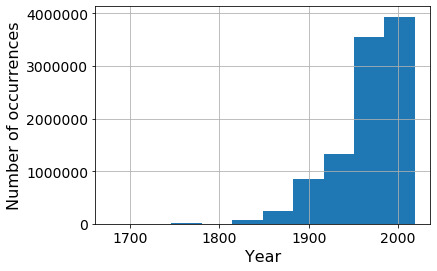
Temporal distribution of metadata for 9,998,299 digital herbarium specimen images from iDigBio and GBIF for *Tracheophyta* (excluding *Polypodiopsida, Poales, Marattiopsida, Pinopsida, Lycopodiopsida* and *Equisetopsida)* with georeference and sampling date available (for more details, refer to section 'Sampling methods').

This search resulted in 9,998,299 specimen images for 182,409 species (2,426,902 images from iDigBio and 7,571,397 images from GBIF). The spatial coverage of preselected images is global across all continents. However, the geo-points located in the oceans indicate problems with georeferences (Fig. 3). The temporal domain of the images mainly covers from 1900 to 2019, with few specimens collected before 1900 (Fig. 4).

**Harmonising names to species level, based on the GBIF backbone taxonomy**: We consolidated the scientific names of species (given by authors of the specimen; see columns 'iDigBio scientificName (given)' and 'GBIF scientificName (given)'), as well as the corresponding accepted scientific names (see columns 'iDigBio scientificName (accepted)' and 'GBIF scientificName (accepted)' in "Digital Herbarium Specimen data", refer to section 'Data resources') provided by iDigBio and GBIF, respectively.

Since scientific names, provided by iDigBio and GBIF for the same specimen, sometimes differ, we additionally provide the corresponding scientific name of the GBIF backbone taxonomy for both, the given scientific names of iDigBio and of GBIF (see columns 'GBIF Backbone Taxonomy scientific name for iDigBio records' and 'GBIF Backbone Taxonomy scientific name for GBIF records').

In order to allow for grouping specimen images per species, we further simplified the scientific names from GBIF backbone taxonomy to binominal names including only genus and species information and ignoring, for example, varieties or subspecies (see column 'Binomial species name for aggregation'). We excluded images for which no species name according to GBIF backbone taxonomy could be identified or where only genus or even broader information was available.

**Figure 5.**
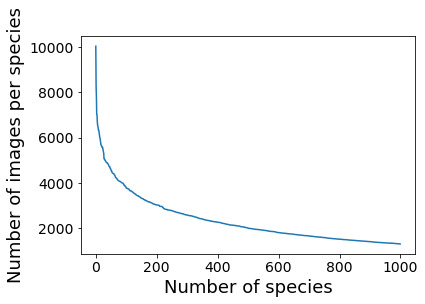
Number of digital herbarium specimen images per species available from iDigBio and GBIF (based on the 9,998,299 images for *Tracheophyta* (excluding *Polypodiopsida, Poales, Marattiopsida, Pinopsida, Lycopodiopsida* and *Equisetopsida)* with georeference and sampling date available (for more details, refer to section 'Sampling methods').

**Selecting appropriate species for our study**: The distribution of images per species has the characteristics of a long-tail distribution: few species with many images, but many species with few images. However, for about 400 of the 182,409 preselected species, iDigBio and GBIF provide more than 2000 images (Fig. 5).

We selected the most promising species for trait data extraction, based on the number of preselected records per species, also considering that sampling sites and dates should be well spread across the species distribution range and in the temporal domain. In addition, the species should have a leaf size and a visible petiole to be easily measurable on the specimen images. Based on these conditions, we selected S*alix bebbiana* Sarg., *Alnus incana* (L.) Moench, *Viola canina* L., *Salix glauca* L., *Chenopodium album* L., *Impatiens capensis* Meerb. and *Solanum dulcamara* L. Table 1 provides the attribution of species and subspecies names received from iDigBio and GBIF to the accepted names in the GBIF taxonomic backbone for the selected species. Table 2 contains the list of datasets downloaded from the GBIF and used in this study.

**Acquisition of image URLs and exclusion of duplicates**: For the selected species, we extracted the Uniform Resource Locators (URLs) from iDigBio and GBIF, under which the herbarium specimen images are stored. Based on the institution code, catalogue number and URL of the specimen images, we identified duplicates within species and excluded them.

**Download of images and final selection for trait measurements**: Each herbarium specimen has a unique combination of institution code and catalogue number to track the specific specimen in different herbaria. We used these two codes to create a unique ID for each specimen (see column 'SpecimenID' in "Digital Herbarium Specimen data", refer to section 'Data resources'). Additionally, we enumerated the SpecimenIDs by image to provide unique ImageIDs in case multiple images were provided for the same specimen. These ImageIDs served as unique image names while downloading the digital herbarium specimen images. As the ImageIDs are based on institution code and catalogue number, they are also helpful for tracking the specific digital herbarium specimen images in the future.

We downloaded 17,383 digital herbarium specimen images for the seven species of interest, based on their URLs using automated Python routines. For each species, it took approximately 4 to 8 hours to download the images. The specific details of the downloaded digital herbarium specimen images are provided in Suppl. material 2 and column descriptions in Table 3.

In addition to removing duplicates from the metadata, based on the institution code, catalogue number and URLs, we found 65 duplicates in the downloaded images (for example, if the digital image of the herbarium specimen were stored in the same or different repositories with different catalogue numbers or institution codes). To systematically identify duplicates, we used the image processing tool fslint, which compares various digital signatures like md5sum and sha1sum (also checks the file size and then checks to ensure they are not hard-linked) and excludes the duplicates.

Some of the remaining images had other problems, such as containing only juvenile leaves (Fig. 6a), incomplete leaf (Fig. 6b), no ruler (Fig. 6c), presence of specimen as a sapling (Fig. 6d), only overlapping leaves (Fig. 6e), no petiole (Fig. 6e) or live photographs (Fig. 6f). In addition to these problematic images, we identified digital herbarium specimens with contorted shapes, as shown in Fig. 7. These images were considered not suitable for measuring leaf traits and discarded (Fig. 2).

**Figure 8.**
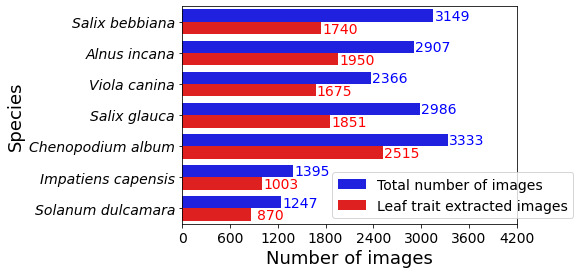
Numbers of images downloaded per species (‘total number of images’) and finally used for trait measurements (‘leaf trait extracted images’) for the seven species of interest.

In total, we discarded 5779 (around 1/3) of the 17,383 downloaded images and retained 11,604 images (around 2/3) for leaf trait extraction (Fig. 8).

The specific details of 17383 digital herbarium specimen images are provided in the file Suppl. material 2 and column description in Table 3.

**Figure 9.**
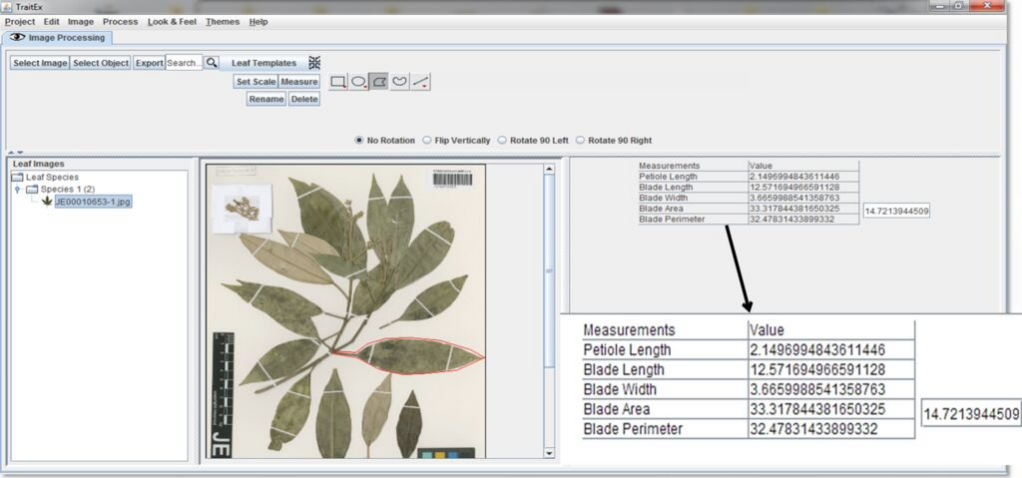
A typical herbarium specimen image in TraitEx. A boundary line (red) has been drawn ‘by hand’ to identify the leaf of interest (‘cropped leaf’). The morphological trait values of that leaf as measured by TraitEx are provided in the upper right corner.

**Figure 10.**
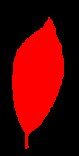
The exact mask of the measured leaf in Fig. 9 from TraitEx workflow.

**Trait measurements**: For the measurement of the quantitative leaf traits - area, perimeter, length and width of individual leaf blades (all provided in "Digital Herbarium Specimen data", refer section 'Data resources'), the TraitEx software (Gaikwad et al. 2019) was used, a semi-automated tool to measure size-related traits on digitised herbarium specimen images. We first uploaded each image into TraitEx and calibrated the length ruler of TraitEx against the ruler bar on the image (Fig. 9, lower-left corner of the specimen image) since the unit length of the ruler will vary from image to image, depending on the image resolution. After calibrating the ruler, the leaf to be measured was selected and an approximate boundary line was drawn 'by hand' around the selected leaf (Fig. 9). The measurement of the exact values of the different traits for the selected leaf within the determined boundary is then done automatically. TraitEx identifies the exact mask of the identified leaf (Fig. 10) and measures the size-related traits on this mask. The results are displayed on the screen (Fig. 9) and saved as a CSV file. The cropped image (inside the boundary line in Fig. 9) and the exact mask of the measured leaf (Fig. 10) is saved for reproducibility. Masks of the leaves contain information on the location of leaves on the digital image and vector data on the boundary lines drawn around the leaf are also saved. Data on masks could be used as training data for leaf segmentation in the future and are available from the corresponding authors upon request.

This measurement process was repeated for each leaf to be measured on a specimen image. We measured the traits on 1 - 5 well-developed leaves per image, depending on the suitability of leaves. On average, it took 10 minutes to measure five leaves on an individual specimen image. It includes the time to import the specimen image into TraitEx, mark up and measure the leaves of interest and visually check the measurements. TraitEx saves the measured leaf trait records as individual CSV files in the folder where the herbarium specimen image is stored. The CSVs files were concatenated after all measurements were finished for a specific species. A detailed description of TraitEx and the measurement process is provided on the TraitEx website.

Finally, we combined the measured leaf trait values with the metadata downloaded from iDigBio and GBIF, based on their ImageIDs (see Suppl. material 1 and section ' Data resources').

**Uncertainties of trait measurements**: Leaf trait measurements from herbarium specimens are associated with uncertainties due to: i) the shrinking of leaves in the preservation process, ii) imaging the herbarium specimen, iii) manual digitisation within the semi-automated workflow of TraitEx and iv) the automated trait measurement of TraitEx.

The uncertainty due to shrinkage during drying is about 3.5 - 15.2 % (Tomaszewski and Górzkowska 2016, Kozlov et al. 2021). To provide estimates for the uncertainties associated with the workflow of processes (ii) scanning and (iv) automated trait extraction with TraitEx, "The authors of the TraitEx software (Triki et al. 2021) selected 20 herbarium specimens of 19 species. As a reference measurement, they measured two leaves per specimen on average using the vernier scale and measured the same leaves with TraitEx on the corresponding digitised herbarium specimens images. The authors then compared the leaf trait values between TraitEx and the manual measurements. The correlation between manual trait measurements and TraitEx measurements was very high (0.998 for leaf length and 0.997 for leaf width) and did not show a bias towards smaller or larger values. However, the uncertainty scales with the reference trait values heteroscedastic of in-situ measurements. The standard error ratio of TraitEx to reference measurements is approximately 1% (leaf length 1.02% and leaf width 0.75%). To estimate uncertainties associated specifically with (iii), the manual digitisation within TraitEx, we measured a single leaf 10 times in a herbarium specimen and repeated the same process on seven different digital herbarium specimens using TraitEx covering all leaf sizes. The uncertainties here have been very small (Table 4).

Therefore, we rounded trait values ("Digital Herbarium Specimen data", refer to section 'Data resources') for leaf width, leaf length and leaf perimeter to a precision of 0.1 cm and leaf area to a precision of 0.1 cm^2^, which corresponds to uncertainties of approximately 1% for leaf length and width for the trait values of seven species we are providing here.

## Geographic coverage

### Description

**Figure 11.**
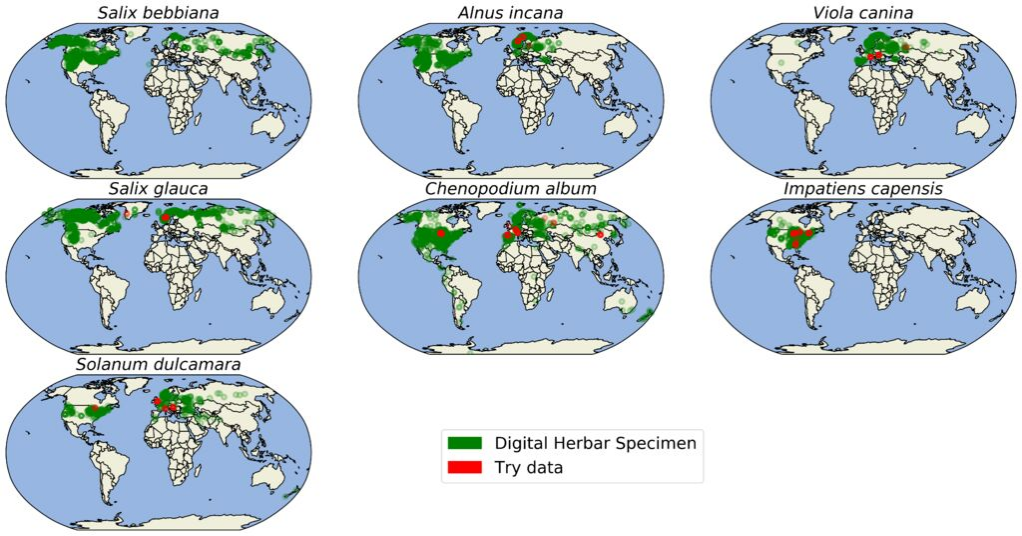
Spatial distributions of measured leaf trait information for *Salix bebbiana* Sarg., *Alnus incana (L.)* Moench, *Viola canina L., Salix glauca L., Chenopodium album L., Impatiens capensis* Meerb. and *Solanum dulcamara L.* from Digital Herbarium Specimen data (green) and the TRY database (red).

Fig. 11 shows the spatial distribution of specimens sampling sites for measured images for the seven species of interest (green dots). We plotted the sampling sites for respective leaf trait measurements in the TRY database (red dots) for comparison. The Latitudes and Longitudes are provided by iDigBio and GBIF; any errors are not the author's responsibility.

## Temporal coverage

**Data range:** 1762-8-03 – 2018-9-12.

### Notes

**Figure 12.**
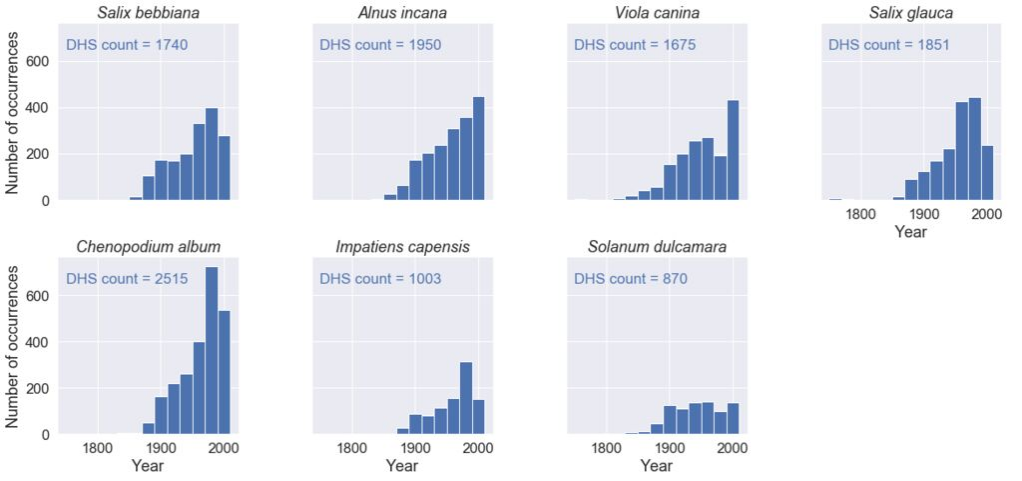
Temporal distributions of measured leaf trait information for *Salix bebbiana* Sarg., *Alnus incana (L.)* Moench, *Viola canina L., Salix glauca L., Chenopodium album L., Impatiens capensis* Meerb. and *Solanum dulcamara L*.

Fig. 12 shows the distribution of specimen sampling years in time for the seven species of interest. Specimen sampling dates back into the 18th century.

## Usage licence

### Usage licence

Other

### IP rights notes

Creative Commons Attribution Waiver (CC-BY) (https://creativecommons.org/licenses/by/4.0)

## Data resources

### Data package title

Digital Herbarium Specimen data

### Resource link

http://doi.org/10.5281/zenodo.4818530

### Alternative identifiers

https://www.try-db.org/TryWeb/Data.php#77

### Number of data sets

1

### Data set 1.

#### Data set name

Digital Herbar Specimen data

#### Data format

comma-separated values

#### Number of columns

27

#### Description

The data package contains 128,036 trait records for leaf-blade area, length, width and perimeter from 32,009 leaves on 11,604 specimen images for the species Salix bebbiana Sarg., Alnus incana (L.) Moench, Viola canina L., Salix glauca L., Chenopodium album L., Impatiens capensis Meerb. and Solanum dulcamara L., including the respective metadata.

In supplementary materials, we provide additional information for each of the 17383 downloaded images: (1) the number of leaves measured on each image or the reason(s) for exclusion of the image from trait measurements (Suppl. material 2); (2) the metadata received from iDigBio and GBIF for each image (Suppl. material 1). For images received via GBIF, we also provide a Table with the references (Table 2).

**Data set 1.** 

Column label	Column description
RowID	Unique identifier for each entry in the data file.
Leaf length in cm	Leaf length of specific entry in cm.
Leaf width in cm	Leaf width of specific entry in cm.
Leaf area in cm^2^	Leaf area of specific entry in cm^2^.
Leaf perimeter in cm	Leaf perimeter of specific entry in cm.
ImageID	Unique identity for each digital herbarium specimen (In the case of multiple entries, measurements are made on different leaves within the same digital herbarium specimen). The binomial name for aggregation is added at the end of the ImageID to ensure each ImageID is unique across the species.
SpecimenID	Provides unique id for each sample (a combination of Institutioncode and Catalognumber), to avoid multiple SpecimenIDs, ImageID is created by enumerating the SpecimenID (occurrence of multiple SpecimenIDs is possible if herbarium specimens are collected from the same sample).
Institutioncode	Code for which Institution the specimen came from.
Catalognumber	Unique identifier of specific specimen in the respective herbarium.
Phylum	Phylum of the species.
Class	Class of the species.
Order	Order of the species.
Family	Family of the species.
iDigBio scientificName (given)	Scientific name extracted from iDigBio metadata.
iDigBio scientificName (accepted)	Accepted scientific name extracted from iDigBio metadata.
GBIF Backbone Taxonomy scientific name for iDigBio records	Scientific name according to the "GBIF Backbone Taxonomy" for iDigBio records.
GBIF scientificName (given)	Scientific name extracted from GBIF metadata.
GBIF scientificName (accepted)	Accepted scientific name extracted from GBIF metadata.
GBIF Backbone Taxonomy scientific name for GBIF records	Scientific name according to the "GBIF Backbone Taxonomy" for GBIF records.
Binomial species name for aggregation	The binomial name for aggregating the scientific names on genus level (Based on the columns 'GBIF Backbone Taxonomy scientific name for GBIF records' and 'GBIF Backbone Taxonomy scientific name for iDigBio records').
Latitude (from iDigBio and GBIF)	Latitude of the collected specimen (extracted from iDigBio and GBIF metadata).
Longitude (from iDigBio and GBIF)	Longitude of the collected specimen (extracted from iDigBio and GBIF metadata).
Sampling date	Sampling date of the collected specimen (extracted from iDigBio and GBIF metadata).
Source	From where the digital herbarium specimen was extracted (iDigBio or GBIF or iDigBio and GBIF). If the source is only iDigBio, the metadata is coming from only IDigBio which means corresponding GBIF entries are updated with the string 'NA' and vice versa.
UUID	Universally Unique IDentifier (UUID) is a unique identifier in iDigBio (this id can be used in the future to request the same data from iDigBio).
GBIFID	GBIFID is a unique identifier in GBIF (this id can be used in the future to request the same data from GBIF)
AccessURL	Link where the digital herbarium specimen is stored.

## Additional information

### Comparison to trait records from plants in natural environments

To test the suitability of leaf trait measurements from digital herbarium specimens, we compared the density distributions of four traits (leaf blade area, length, width and perimeter) measured on the herbarium specimen images against records based on standard measurement protocols from the TRY database on plant traits (Kattge et al. 2020, Kattge et al. 2011). In this context, leaf area is defined as the projected area of an individual leaf (Pérez-Harguindeguy et al. 2013), which matches the measurements on the specimen images. However, the standard measurement protocol recommends measuring about ten mature leaves from the sunlit part of the canopy for each site in the natural environment and during the full flowering period (Pérez-Harguindeguy et al. 2013). In the case of a herbarium specimen, this selection cannot be guaranteed. There might be various sampling biases:

- Herbarium specimens are often collected near roadsides, populated areas or universities
- They are collected during campaigns that might be outside the flowering period
- They might be taken from parts of the canopy which are not in full sunlight.

The sampled individual also might not be representative of the species. In the TRY database, sometimes all measurements are reported, sometimes only the average per individual and sometimes the average per species and site. We here compare the averages per specimen image (on average, three leaves measured per image) to trait records derived from the TRY database (Kattge et al. 2020, Kattge et al. 2011) in case of TRY not distinguishing individual measurements from average values.

**Figure 13.**
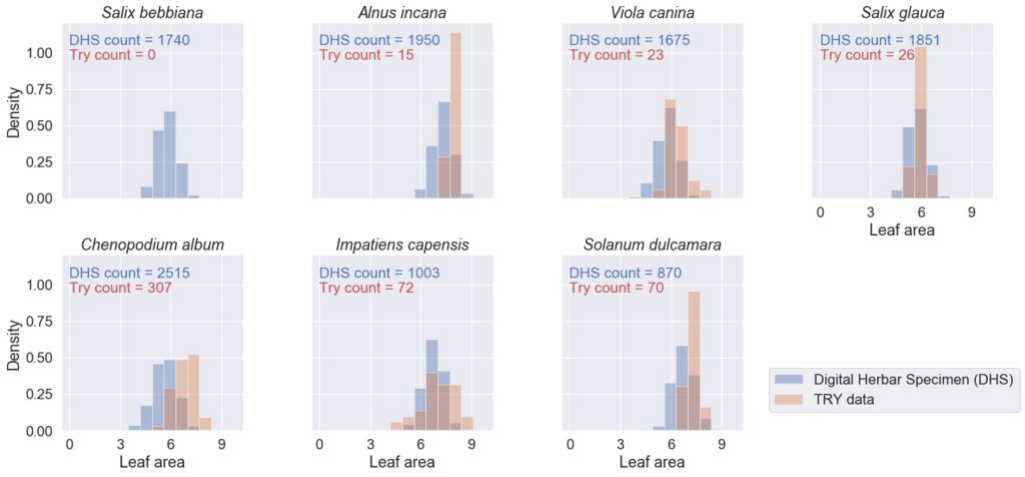
Comparison of the density distributions of leaf blade area (mm^2^, log-transformed) from herbarium specimen images to trait records, derived from the TRY database (representing trait measurements from life individuals by standard protocols) for the seven species of interest.

**Figure 14.**
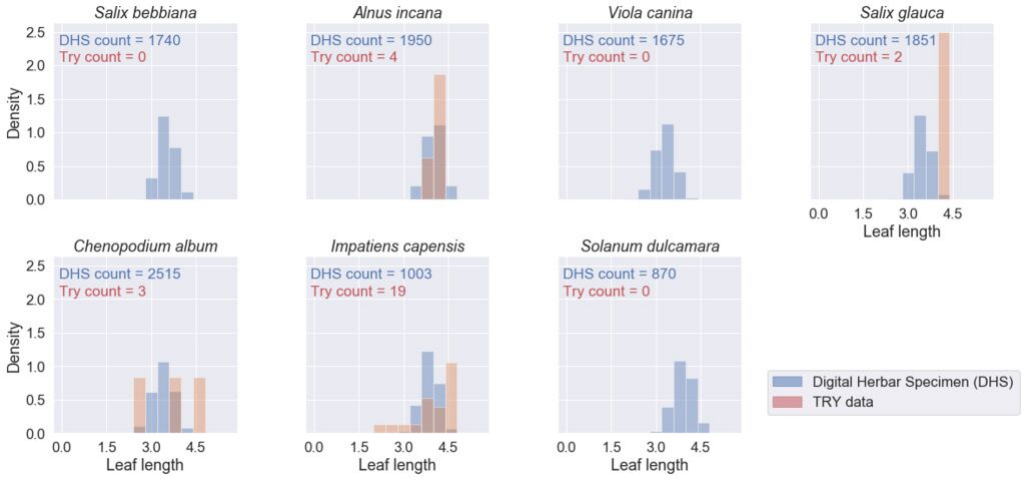
Comparison of the density distributions of leaf blade length (mm, log-transformed) from herbarium specimen images to trait records, derived from the TRY database (representing trait measurements from life individuals by standard protocols) for the seven species of interest.

**Figure 15.**
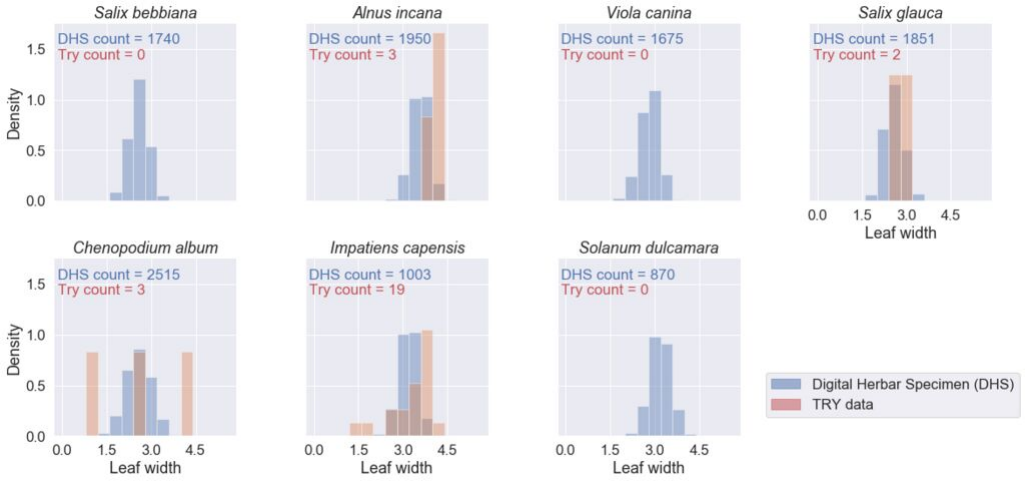
Comparison of the density distributions of leaf blade width (mm, log-transformed) from herbarium specimen images to trait records, derived from the TRY database (representing trait measurements from life individuals by standard protocols) for the seven species of interest.

**Figure 16.**
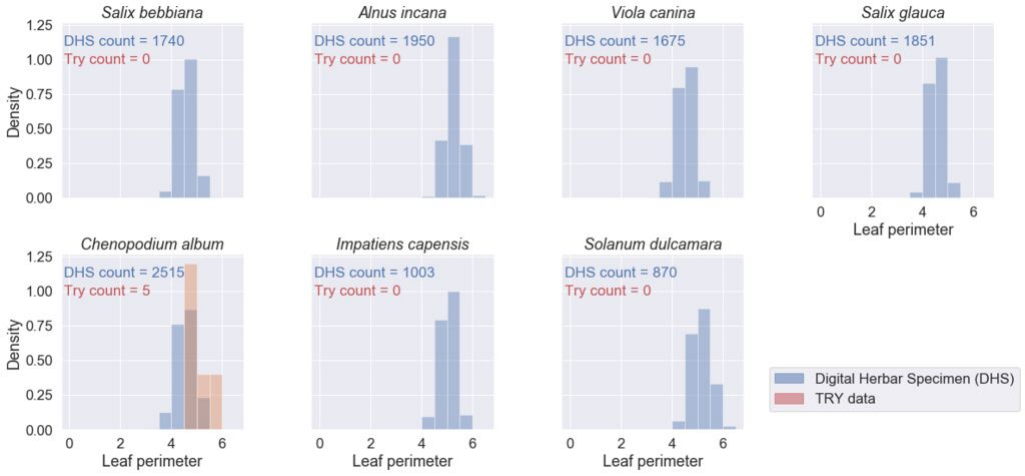
Comparison of the density distributions of leaf blade perimeter (mm, log-transformed) from herbarium specimen images to trait records, derived from the TRY database (representing trait measurements from life individuals by standard protocols) for the seven species of interest.

We retrieved 573 trait records for the four traits and the seven species of interest from the TRY database - compared to 46,416 mean trait values from 11,604 specimen images. For several species-trait combinations, the TRY database contained zero records (see Figs 13, 14, 15, 16). The spatial distribution of the records from the TRY database was minimal compared to the spatial range of the measured herbarium specimens (see Fig. 11). The density distributions of trait records, based on herbarium specimens, follow an approximately normal distribution (after log-transformation) for all measured leaf traits and species. The range of trait values from the TRY database (if there are some) is overlapping the range of trait values from the herbarium specimen images for all trait-species combinations (see Figs 13, 14, 15, 16). However, if the number of trait records derived from the TRY database were sufficient for a more detailed comparison, the density distributions, based on specimen images, show a small, but consistent bias to smaller values (e.g. leaf area, one of the best covered continuous traits in the TRY database). We tend to explain this by the differences in the measurement protocols: the standard protocol recommends selecting ten mature leaves from the sunlit canopy, while we selected up to five leaves from a specimen image. Selecting several leaves from a specimen image includes a higher risk of sampling smaller, not fully mature leaves and leaf sizes might become smaller in the drying process while making digital herbarium specimens (Tomaszewski and Górzkowska 2016). However, this small, but rather consistent bias still needs to be addressed, based on comprehensive sampling across more species.

### Discussion

Millions of digitised herbarium specimen images have become available during recent years and the numbers are expected to rise. Based on the metadata provided via the iDigBio and GBIF data portals, we were able to filter the images along with taxonomy and required additional information - georeference and sampling date. This enabled us to constrain the 21.9 million and 31.6 million herbarium specimen images available via iDigBio and GBIF (accessed date December 2020) for plants in the Phylum *Tracheophyta* to about 10 million images with sufficient additional information. Based on this pre-selection, we identified seven species most promising for data extraction and analysis in the context of this pilot study. We finally downloaded 17,383 images. We had to discard 5779 of the downloaded images (about 1/3) because of duplications or other problems not visible in the metadata. Nevertheless, we finally retained about 900 to 2500 images per species, covering broad species distribution ranges and dating back to the 19th century. Extracting trait values on average about three leaves per image, the final trait dataset contains 128,036 records for leaf area, length, width and perimeter from 32,009 leaves. Separate uncertainty analyses and the comparison to leaf traits measured in natural environments following standard protocols indicate the validity of the trait values extracted from the herbarium specimen images. The dataset provided here increases the number of trait records for the seven selected species compared to other available trait data by up to three orders of magnitude and justifies hope for substantially improved analyses of trait variation within species and across space and time.

However, this pilot study also identified two bottlenecks towards extracting trait records for a more comprehensive number of species. The first bottleneck is the time needed to download the digitised herbarium specimen images. Even though this process was automated using Python scripts, it took approximately 5 hours for 2000 images. This was acceptable for our pilot study, based on seven species, but may be a problem for measurement campaigns across a more comprehensive number of species and images. Every improvement to better select the images appropriate for measurements, without downloading the images or/and speed up the download for individual images, will therefore substantially improve the opportunity for comprehensive trait data extraction from millions of herbarium specimen images. The other bottleneck is the time and the human input needed to measure trait values using the TraitEx software. TraitEx is a semi-automated tool and it takes about 10 minutes to extract, check and save the trait measurements per specimen image. This was manageable for our use case with a constrained number of 17,383 images suitable for trait measurements. However, for comprehensive measurement campaigns across all appropriate images, potentially covering millions of images, a fully automated tool is needed, which seamlessly combines robust automated detection of suitable leaves with the precise measurement of size-related traits.

## Supplementary Material

260ECA78-3D27-5F68-AD9E-72A5A181EDBD 10.3897/BDJ.9.e69806.suppl1 Supplementary material 1 Metadata from iDigBio and GBIF Data type comma-separated values Brief description This file contains the metadata of the 17383 digital herbarium specimen images from iDigBio and GBIF for selected seven species. File: oo_548998.csv https://binary.pensoft.net/file/548998 Vamsi Krishna Kommineni, Susanne Tautenhahn, Pramod Baddam, Jitendra Gaikwad, Barbara Wieczorek, Abdelaziz Triki, Jens Kattge

F9029DEB-A4B1-50CA-9EC8-3F08DA0A1710 10.3897/BDJ.9.e69806.suppl2 Supplementary material 2 Information of digital herbarium specimen images with different kinds of problems Data type comma-separated values (csv) Brief description The columns in the data file 'Image', 'Number of leaves measured', 'Remarks_1', 'Remarks_2', and 'Ruler' explains different kinds of information about the corresponding digital herbarium specimen record. For more information about the data columns, please refer to Table 2. Only 17249 herbarium specimen images data are available in this file; the remaining 134 data points were discarded while dropping the duplicates.
If all the columns 'Image', 'Number of leaves measured', 'Remarks_1', 'Remarks_2' and 'Ruler' contain string 'NA', meaning AccessURL of the corresponding record is not responded or not reachable while downloading the images.
If 'Number of leaves measured' column is 'NA', then one of the columns 'Image', 'Remarks_1', 'Remarks_2', and 'Ruler' are updated accordingly, meaning the trait measurement is not possible. File: oo_549000.csv https://binary.pensoft.net/file/549000 Vamsi Krishna Kommineni, Susanne Tautenhahn, Pramod Baddam, Jitendra Gaikwad, Barbara Wieczorek, Abdelaziz Triki, Jens Kattge

## Figures and Tables

**Figure 6a.**
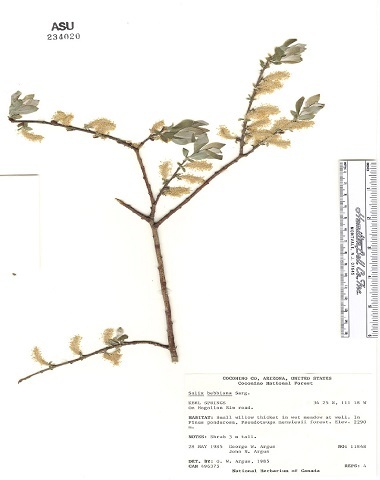
Only juvenile leaves: only small leaves along with small flowers.

**Figure 6b.**
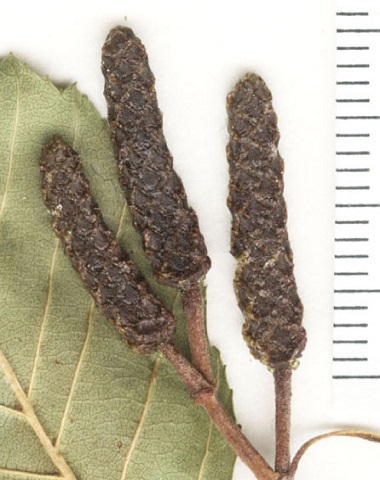
Incomplete leaf: no complete leaf on the specimen.

**Figure 6c.**
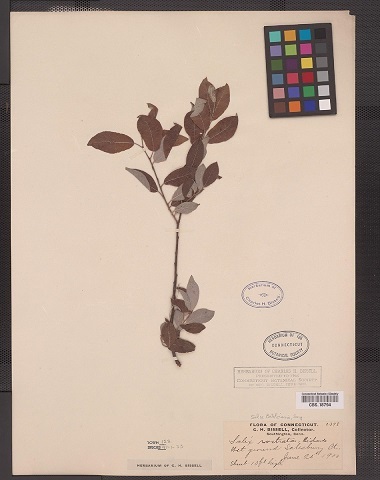
Missing ruler

**Figure 6d.**
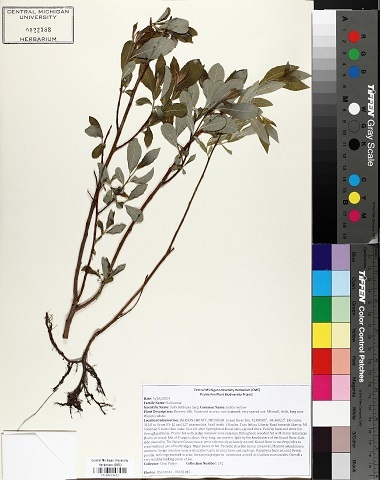
Sapling (juvenile plant)

**Figure 6e.**
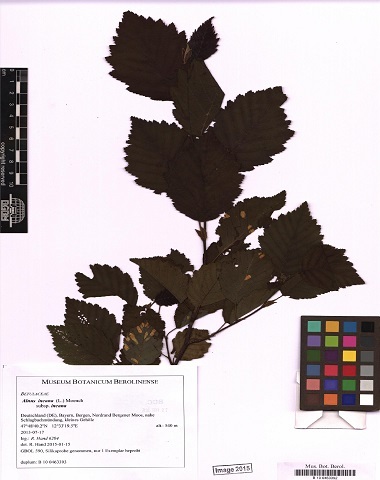
All leaves are overlapping

**Figure 6f.**
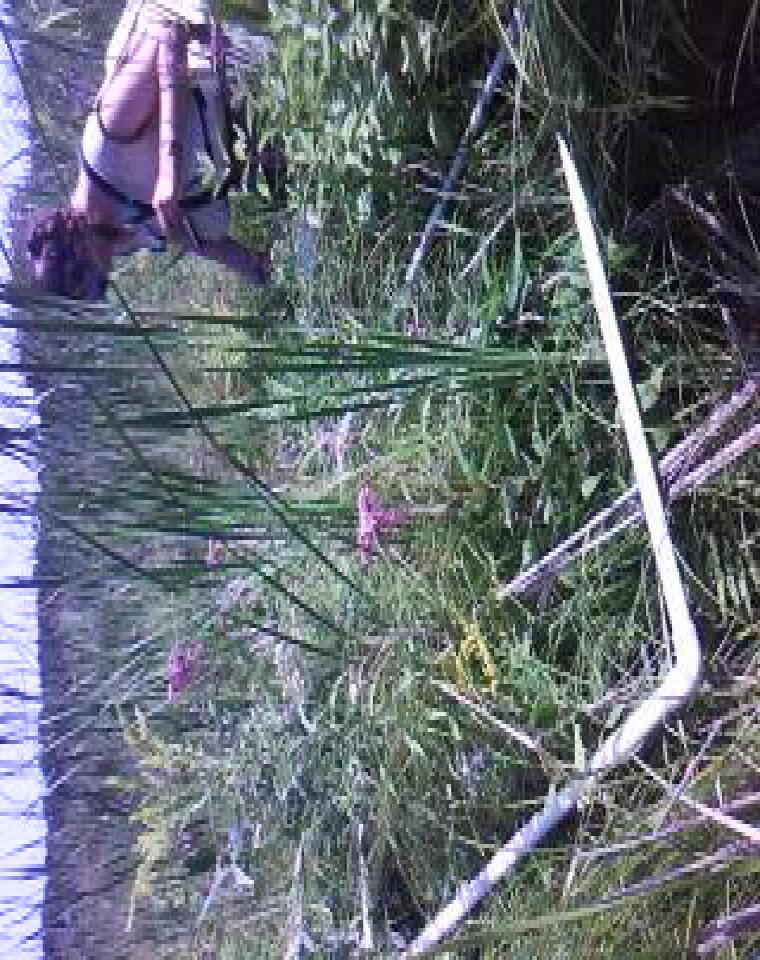
Live photograph: this is not a digitised herbarium specimen.

**Figure 7a.**
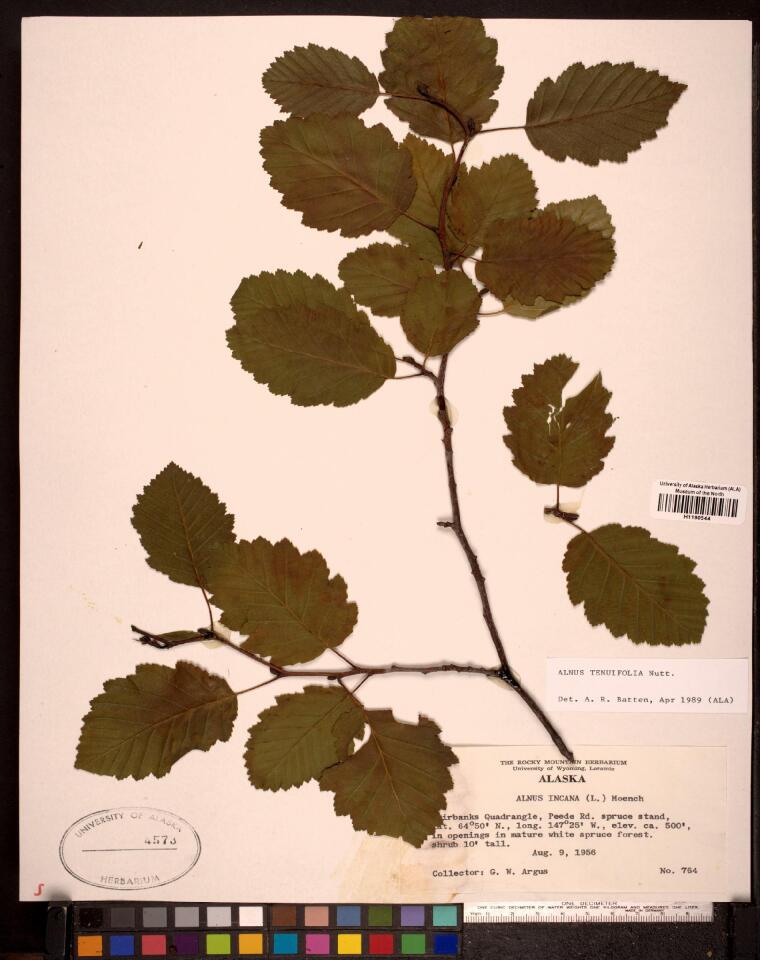
Horizontal orientation of the digital herbarium specimen: we considered only images with horizontal orientation for trait measurements.

**Figure 7b.**
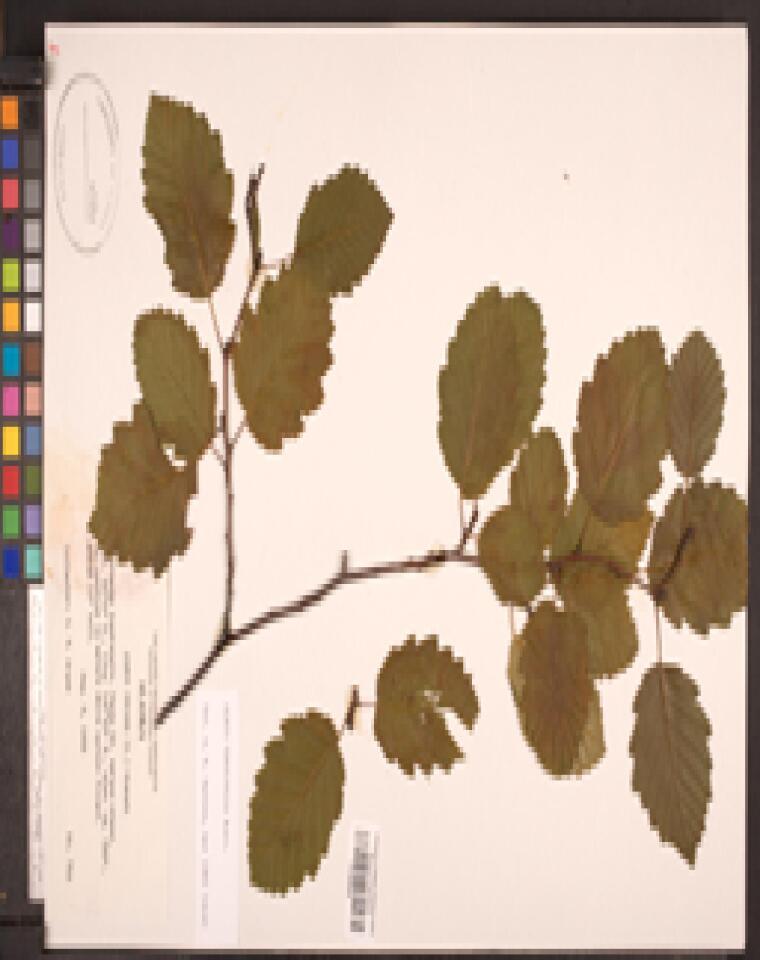
Vertical orientation of the digital herbarium specimen: the image represents the same specimen and image, but in a different orientation with contorted shapes. We removed all images with the vertical orientation of herbarium specimens because the images were potentially contorted by the resize process while downloading.

**Table 1.** Attribution of (given) scientific names to accepted species names, based on the GBIF backbone taxonomy for the seven species of interest: *Salix bebbiana* Sarg., *Alnus incana* (L.) Moench, *Viola canina* L., *Salix glauca* L., *Chenopodium album* L., *Impatiens capensis* Meerb. and *Solanum dulcamara* L.

**Given scientificName (from iDigBio or GBIF)**	**Accepted scientificName (from iDigBio or GBIF)**	**Scientific name according to GBIF Backbone Taxonomy**	**Binomial species name for aggregation**
*salix bebbiana*	*Salix bebbiana*	*Salix bebbiana* Sarg.	*Salix bebbiana*
*salix eriocephala* var. ligulifolia	Salix eriocephala var. ligulifolia	Salix eriocephala var. ligulifolia (C.R.Ball) Dorn	*Salix bebbiana*
*salix planifolia*	*Salix planifolia*	*Salix planifolia* Pursh	*Salix bebbiana*
*salix monticola*	*Salix monticola*	*Salix monticola* Bebb	*Salix bebbiana*
*salix scoulerana*	*Salix scoulerana*	*Salix scoulerana* Barratt ex Hook.	*Salix bebbiana*
*salix bebbiana* sarg.	*Salix bebbiana* Sarg.	*Salix bebbiana* Sarg.	*Salix bebbiana*
*salix bebbiana* var. bebbiana	Salix bebbiana var. bebbiana	Salix bebbiana var. bebbiana	*Salix bebbiana*
*salix livida* var. occidentalis (andersson) a. gray	Salix livida var. occidentalis (Andersson) A. Gray	Salix livida var. occidentalis (Andersson) A.Gray	*Salix bebbiana*
*salix bebbiana* var. perrostrata (rydb.) c.k.schneid.	Salix bebbiana var. perrostrata (Rydb.) C.K.Schneid.	Salix bebbiana var. perrostrata (Rydb.) C.K.Schneid.	*Salix bebbiana*
*salix perrostrata* rydb.	*Salix perrostrata* Rydb.	*Salix perrostrata* Rydb.	*Salix bebbiana*
*salix rostrata* richardson	*Salix rostrata* Richardson	*Salix rostrata* Richardson	*Salix bebbiana*
*salix bebbiana* var. depilis raup	Salix bebbiana var. depilis Raup	Salix bebbiana var. depilis Raup	*Salix bebbiana*
*alnus incana* subsp. rugosa (du roi) r.t.clausen	Alnus incana subsp. rugosa (Du Roi) R.T.Clausen	Alnus incana subsp. rugosa (Du Roi) R.T.Clausen	*Alnus incana*
*alnus incana* subsp. rugosa (du roi) clausen	Alnus incana subsp. rugosa (Du Roi) Clausen	Alnus incana subsp. rugosa (Du Roi) R.T.Clausen	*Alnus incana*
*alnus incana* subsp. rugosa	Alnus incana subsp. rugosa	Alnus incana subsp. rugosa (Du Roi) R.T.Clausen	*Alnus incana*
*alnus incana* ssp. rugosa	Alnus incana ssp. rugosa	Alnus incana subsp. rugosa (Du Roi) R.T.Clausen	*Alnus incana*
*alnus incana* (l.) moench subsp. rugosa (du roi) r.t.clausen	Alnus incana (L.) Moench subsp. rugosa (Du Roi) R.T.Clausen	Alnus incana subsp. rugosa (Du Roi) R.T.Clausen	*Alnus incana*
*alnus incana* subsp. tenuifolia (nutt.) breitung	Alnus incana subsp. tenuifolia (Nutt.) Breitung	Alnus incana subsp. tenuifolia (Nutt.) Breitung	*Alnus incana*
*alnus incana* subsp. tenuifolia	Alnus incana subsp. tenuifolia	Alnus incana subsp. tenuifolia (Nutt.) Breitung	*Alnus incana*
*alnus incana* (l.) moench subsp. tenuifolia (nutt.) breitung	Alnus incana (L.) Moench subsp. tenuifolia (Nutt.) Breitung	Alnus incana subsp. tenuifolia (Nutt.) Breitung	*Alnus incana*
*alnus incana* ssp. *kolaënsis*	*Alnus incana* ssp. *kolaënsis*	Alnus incana subsp. kolaensis (Orlova) Á.Löve & D.Löve	*Alnus incana*
*alnus incana* (l.) moench	*Alnus incana* (L.) Moench	*Alnus incana* (L.) Moench	*Alnus incana*
*alnus incana*	*Alnus incana*	*Alnus incana* (L.) Moench	*Alnus incana*
*alnus incana* subsp. crispa [ined.]	*Alnus incana* subsp. Crispa [ined.]	*Alnus incana* (L.) Moench	*Alnus incana*
*alnus rugosa*	*Alnus rugosa*	*Alnus rugosa* hort. ex Regel, 1868	*Alnus incana*
*alnus rugosa* (du roi) spreng.	*Alnus rugosa* (Du Roi) Spreng.	*Alnus rugosa* hort. ex Regel, 1868	*Alnus incana*
*alnus incana* ssp. incana	Alnus incana ssp. incana	Alnus incana subsp. incana	*Alnus incana*
*alnus incana* var. occidentalis (dippel) c.l.hitchc.	Alnus incana var. occidentalis (Dippel) C.L.Hitchc.	Alnus incana var. occidentalis (Dippel) Hitchc.	*Alnus incana*
*alnus incana* var. occidentalis	Alnus incana var. occidentalis	Alnus incana var. occidentalis (Dippel) Hitchc.	*Alnus incana*
*alnus incana* subsp. rugosa var. occidentalis (dippel) c.l.hitchc.	Alnus incana subsp. rugosa var. occidentalis (Dippel) C.L.Hitchc.	Alnus incana var. occidentalis (Dippel) Hitchc.	*Alnus incana*
*alnus tenuifolia* nutt.	*Alnus tenuifolia* Nutt.	*Alnus tenuifolia* Nutt.	*Alnus incana*
*alnus tenuifolia*	*Alnus tenuifolia*	*Alnus tenuifolia* Nutt.	*Alnus incana*
*alnus incana* (l.) moench ssp. rugosa (du roi) clausen	Alnus incana (L.) Moench ssp. rugosa (Du Roi) Clausen	Alnus incana subsp. rugosa (Du Roi) R.T.Clausen	*Alnus incana*
*alnus incana* ssp. tenuifolia	Alnus incana ssp. tenuifolia	Alnus incana subsp. tenuifolia (Nutt.) Breitung	*Alnus incana*
*alnus incana* var. virescens	Alnus incana var. virescens	Alnus incana var. virescens S.Watson	*Alnus incana*
*alnus incana* (l.) moench ssp. tenuifolia (nutt.) breitung	Alnus incana (L.) Moench ssp. tenuifolia (Nutt.) Breitung	Alnus incana subsp. tenuifolia (Nutt.) Breitung	*Alnus incana*
*alnus rugosa* var. americana	Alnus rugosa var. americana	Alnus rugosa var. americana (Regel) Fernald	*Alnus incana*
*alnus incana* (l.) moench subsp. incana	Alnus incana (L.) Moench subsp. incana	Alnus incana subsp. incana	*Alnus incana*
*viola canina*	*Viola canina*	*Viola canina* L.	*Viola canina*
*viola canina* l.	*Viola canina* L.	*Viola canina* L.	*Viola canina*
*viola canina* l. 'white butterfly'	*Viola canina* L. 'White Butterfly'	*Viola canina* L.	*Viola canina*
*viola canina* ssp. montana	Viola canina ssp. montana	Viola canina subsp. montana (L.) Lange	*Viola canina*
*viola canina* ssp. canina	Viola canina ssp. canina	Viola canina subsp. canina	*Viola canina*
*viola canina* l. subsp. montana (l.) hartm.	Viola canina L. subsp. montana (L.) Hartm.	Viola canina subsp. montana (L.) Lange	*Viola canina*
*viola nummularifolia* all.	*Viola nummularifolia* All.	*Viola nummularifolia* F.W.Schmidt	*Viola canina*
*viola longipes* nutt.	*Viola longipes* Nutt.	*Viola longipes* Nutt.	*Viola canina*
*salix glauca*	*Salix glauca*	*Salix glauca* L.	*Salix glauca*
*salix glauca* ssp. stipulifera	Salix glauca ssp. stipulifera	Salix glauca subsp. stipulifera (Flod. ex Häyrén) Hiitonen	*Salix glauca*
*salix glauca* l.	*Salix glauca* L.	*Salix glauca* L.	*Salix glauca*
*salix glauca* var.	*Salix glauca* var.	*Salix glauca* L.	*Salix glauca*
*salix glauca/brachycarpa*	*Salix glauca/brachycarpa*	*Salix glauca* L.	*Salix glauca*
*salix pseudolapponum* seemen	*Salix pseudolapponum* Seemen	*Salix pseudolapponum* Seem.	*Salix glauca*
*salix glauca* var. acutifolia (hook.) c. k. schneid.	Salix glauca var. acutifolia (Hook.) C. K. Schneid.	Salix glauca var. acutifolia (Hook.) C.K.Schneid.	*Salix glauca*
*salix glauca* var. acutifolia	Salix glauca var. acutifolia	Salix glauca var. acutifolia (Hook.) C.K.Schneid.	*Salix glauca*
*salix glauca* subsp. glauca var. acutifolia	Salix glauca subsp. glauca var. acutifolia	Salix glauca var. acutifolia (Hook.) C.K.Schneid.	*Salix glauca*
*salix glauca* ssp. glauca	Salix glauca ssp. glauca	Salix glauca subsp. glauca	*Salix glauca*
*salix glauca* l. subsp. glauca	Salix glauca L. subsp. glauca	Salix glauca subsp. glauca	*Salix glauca*
*salix glauca* var. cordifolia (pursh) dorn	Salix glauca var. cordifolia (Pursh) Dorn	Salix glauca var. cordifolia (Pursh) Dorn	*Salix glauca*
*salix glauca* var. cordifolia	Salix glauca var. cordifolia	Salix glauca var. cordifolia (Pursh) Dorn	*Salix glauca*
*salix labradorica*	*Salix labradorica*	*Salix labradorica* Rydb.	*Salix glauca*
*salix glauca* subsp. callicarpaea	Salix glauca subsp. callicarpaea	Salix glauca subsp. callicarpaea (Trautv.) Böcher	*Salix glauca*
*salix glauca* subsp. callicarpaea (trautv.) böcher	Salix glauca subsp. callicarpaea (Trautv.) Böcher	Salix glauca subsp. callicarpaea (Trautv.) Böcher	*Salix glauca*
*salix anamesa* c. k. schneid.	*Salix anamesa* C. K. Schneid.	*Salix anamesa* C.K.Schneid.	*Salix glauca*
*salix glauca* var. villosa andersson	Salix glauca var. villosa Andersson	Salix glauca var. villosa (Hook.) Andersson	*Salix glauca*
*salix glauca* subsp. villosa	Salix glauca subsp. villosa	Salix glauca subsp. villosa (Hook.) A.E.Murray	*Salix glauca*
*salix glauca* var. villosa	Salix glauca var. villosa	Salix glauca var. villosa (Hook.) Andersson	*Salix glauca*
*salix glauca* subsp. glauca var. villosa	Salix glauca subsp. glauca var. villosa	Salix glauca var. villosa (Hook.) Andersson	*Salix glauca*
*salix glauca* var. villosa (hook.) andersson	Salix glauca var. villosa (Hook.) Andersson	Salix glauca var. villosa (Hook.) Andersson	*Salix glauca*
*salix glaucops* andersson	*Salix glaucops* Andersson	*Salix glaucops* Andersson	*Salix glauca*
*salix desertorum* richardson	*Salix desertorum* Richardson	*Salix desertorum* Richardson	*Salix glauca*
*salix wyomingensis* rydb.	*Salix wyomingensis* Rydb.	*Salix wyomingensis* Rydb.	*Salix glauca*
*salix glauca* var. glauca	Salix glauca var. glauca	Salix glauca var. glauca	*Salix glauca*
*salix glauca* l. f. appendiculata	Salix glauca L. f. appendiculata	Salix glauca var. appendiculata (Vahl) Wahlenb.	*Salix glauca*
*salix glauca* var. macounii (rydb.) b.boivin	Salix glauca var. macounii (Rydb.) B.Boivin	Salix glauca var. macounii (Rydb.) B.Boivin	*Salix glauca*
*salix glauca* var. glabrescens c.k.schneid.	Salix glauca var. glabrescens C.K.Schneid.	Salix glauca var. glabrescens (Andersson) C.K.Schneid.	*Salix glauca*
*salix glauca* var. glabrescens	Salix glauca var. glabrescens C.K.Schneid.	Salix glauca var. glabrescens (Andersson) C.K.Schneid.	*Salix glauca*
*salix cordifolia* pursh	*Salix cordifolia* Pursh	*Salix cordifolia* Banks ex Pursh	*Salix glauca*
*salix glauca* var. aliceae	Salix glauca var. aliceae	Salix glauca var. aliceae C.R.Ball	*Salix glauca*
*salix glauca* var. stenolepis	Salix glauca var. stenolepis	Salix glauca var. stenolepis (Flod.) Polunin	*Salix glauca*
*salix callicarpaea* trautv.	*Salix callicarpaea* Trautv.	*Salix callicarpaea* Trautv.	*Salix glauca*
*salix glauca* subsp. acutifolia	Salix glauca subsp. acutifolia	Salix glauca subsp. acutifolia (Hook.) Hultén	*Salix glauca*
*salix glauca* var. perstipula raup	Salix glauca var. perstipula Raup	Salix glauca var. perstipula Raup	*Salix glauca*
*salix glauca* var. perstipula	Salix glauca var. perstipula	Salix glauca var. perstipula Raup	*Salix glauca*
*salix cordifolia* pursh var. callicarpea	Salix cordifolia Pursh var. callicarpea	Salix cordifolia var. callicarpaea (Trautv.) Fernald	*Salix glauca*
*salix cordifolia* var. callicarpaea (trautv.) fernald	Salix cordifolia var. callicarpaea (Trautv.) Fernald	Salix cordifolia var. callicarpaea (Trautv.) Fernald	*Salix glauca*
*salix glauca* var. stipulata	Salix glauca var. stipulata	Salix glauca var. stipulata Floderus	*Salix glauca*
*salix glauca* subsp. desertorum	Salix glauca subsp. desertorum	Salix glauca subsp. desertorum (Richardson) Hultén	*Salix glauca*
*salix glauca* var. callicarpaea	Salix glauca var. callicarpaea	Salix glauca var. callicarpaea (Pursh) Dorn	*Salix glauca*
*salix glauca* var. acutifolia (hook.) c.k. schneid.	Salix glauca var. acutifolia (Hook.) C.K. Schneid.	Salix glauca var. acutifolia (Hook.) C.K.Schneid.	*Salix glauca*
*chenopodium album*	*Chenopodium album*	*Chenopodium album* L.	*Chenopodium album*
*chenopodium album* l.	*Chenopodium album* L.	*Chenopodium album* L.	*Chenopodium album*
*chenopodium* cf. album	Chenopodium cf. album	*Chenopodium album* L.	*Chenopodium album*
*chenopodium album* zz auct. var. striatum krašan	Chenopodium album ZZ auct. var. striatum Krašan	*Chenopodium album* L.	*Chenopodium album*
*chenopodium missouriense* aellen	*Chenopodium missouriense* Aellen	*Chenopodium missouriense* Aellen	*Chenopodium album*
*chenopodium missouriense*	*Chenopodium missouriense*	*Chenopodium missouriense* Aellen	*Chenopodium album*
*chenopodium* cf. *missouriense*	*Chenopodium* cf. *Missouriense*	*Chenopodium missouriense* Aellen	*Chenopodium album*
*chenopodium lanceolatum*	*Chenopodium lanceolatum*	*Chenopodium lanceolatum* Muhl. ex Willd.	*Chenopodium album*
*chenopodium lanceolatum* muhl. ex willd.	*Chenopodium lanceolatum* Muhl. ex Willd.	*Chenopodium lanceolatum* Muhl. ex Willd.	*Chenopodium album*
*chenopodium album* var. album	Chenopodium album var. album	Chenopodium album var. album	*Chenopodium album*
*chenopodium paganum*	*Chenopodium paganum*	*Chenopodium paganum* Rchb.	*Chenopodium album*
*chenopodium paganum* rchb.	*Chenopodium paganum* Rchb.	*Chenopodium paganum* Rchb.	*Chenopodium album*
*chenopodium album* var. *missouriense*	Chenopodium album var. missouriense	Chenopodium album var. missouriense (Aellen) Bassett & Crompton	*Chenopodium album*
*chenopodium viride* l.	*Chenopodium viride* L.	*Chenopodium* viride L.	*Chenopodium album*
*chenopodium album* l. subsp. album	Chenopodium album L. subsp. album	Chenopodium album subsp. album	*Chenopodium album*
*chenopodium suecicum*	*Chenopodium suecicum*	*Chenopodium suecicum* Murr	*Chenopodium album*
*chenopodium album* var. lanceolatum	Chenopodium album var. lanceolatum	Chenopodium album var. lanceolatum (Muhl.) Coss. & Germ.	*Chenopodium album*
*chenopodium paganum* auct. non reichenb.	*Chenopodium paganum* auct. Non Reichenb.	*Chenopodium paganum* Rchb.	*Chenopodium album*
*chenopodium album* var. viride	Chenopodium album var. viride	Chenopodium album var. viride (L.) Moq.	*Chenopodium album*
*chenopodium album* l. var. integerrimum s. f. gray	Chenopodium album L. var. integerrimum S. F. Gray	*Chenopodium album* L.	*Chenopodium album*
*chenopodium album* var. album l.	Chenopodium album var. album L.	Chenopodium album var. album	*Chenopodium album*
*chenopodium album* var. stevensii	Chenopodium album var. stevensii	Chenopodium album var. stevensii Aellen	*Chenopodium album*
*solanum dulcamara*	*Solanum dulcamara*	*Solanum dulcamara* L.	*Solanum dulcamara*
*solanum dulcamara* l.	*Solanum dulcamara* L.	*Solanum dulcamara* L.	*Solanum dulcamara*
*solanum dulcamara* var. dulcamara	Solanum dulcamara var. dulcamara	Solanum dulcamara var. dulcamara	*Solanum dulcamara*
*impatiens capensis*	*Impatiens capensis*	*Impatiens capensis* Meerb.	*Impatiens capensis*
*Salix bebbiana* Sarg.	*Salix bebbiana* Sarg.	*Salix bebbiana* Sarg.	*Salix bebbiana*
Salix eriocephala var. ligulifolia (C.R.Ball) R.D.Dorn	*Salix ligulifolia* C.R.Ball ex C.K.Schneid.	Salix eriocephala var. ligulifolia (C.R.Ball) Dorn	*Salix bebbiana*
*Salix planifolia* Pursh	*Salix planifolia* Pursh	*Salix planifolia* Pursh	*Salix bebbiana*
*Salix monticola* Bebb	*Salix monticola* Bebb	*Salix monticola* Bebb	*Salix bebbiana*
*Salix scoulerana* Barratt ex Hook.	*Salix scouleriana* Barratt ex Hook.	*Salix scoulerana* Barratt ex Hook.	*Salix bebbiana*
Salix bebbiana var. bebbiana	*Salix bebbiana* Sarg.	Salix bebbiana var. bebbiana	*Salix bebbiana*
Salix livida var. occidentalis (Andersson) A.Gray	*Salix bebbiana* Sarg.	Salix livida var. occidentalis (Andersson) A.Gray	*Salix bebbiana*
Salix bebbiana var. perrostrata (Rydb.) C.K.Schneid.	*Salix bebbiana* Sarg.	Salix bebbiana var. perrostrata (Rydb.) C.K.Schneid.	*Salix bebbiana*
*Salix perrostrata* Rydb.	*Salix bebbiana* Sarg.	*Salix perrostrata* Rydb.	*Salix bebbiana*
*Salix rostrata* Richards.	*Salix bebbiana* Sarg.	*Salix rostrata* Richardson	*Salix bebbiana*
Salix bebbiana var. depilis Raup	*Salix bebbiana* Sarg.	Salix bebbiana var. depilis Raup	*Salix bebbiana*
Alnus incana subsp. rugosa (Du Roi) R.T.Clausen	Alnus incana subsp. rugosa (Du Roi) R.T.Clausen	Alnus incana subsp. rugosa (Du Roi) R.T.Clausen	*Alnus incana*
Alnus incana subsp. tenuifolia (Nutt.) Breitung	Alnus incana subsp. tenuifolia (Nutt.) Breitung	Alnus incana subsp. tenuifolia (Nutt.) Breitung	*Alnus incana*
Alnus incana subsp. kolaensis (Orlova) Á.Löve & D.Löve	Alnus incana subsp. kolaensis (Orlova) Á.Löve & D.Löve	Alnus incana subsp. kolaensis (Orlova) Á.Löve & D.Löve	*Alnus incana*
*Alnus incana* (L.) Moench	*Alnus incana* (L.) Moench	*Alnus incana* (L.) Moench	*Alnus incana*
*Alnus rugosa* (Du Roi) Spreng.	Alnus incana subsp. rugosa (Du Roi) R.T.Clausen	*Alnus rugosa* (Du Roi) Spreng.	*Alnus incana*
Alnus incana subsp. incana	Alnus incana subsp. incana	Alnus incana subsp. incana	*Alnus incana*
Alnus incana var. occidentalis (Dippel) Hitchc.	Alnus incana subsp. tenuifolia (Nutt.) Breitung	Alnus incana var. occidentalis (Dippel) Hitchc.	*Alnus incana*
*Alnus tenuifolia* Nutt.	Alnus incana subsp. tenuifolia (Nutt.) Breitung	*Alnus tenuifolia* Nutt.	*Alnus incana*
Alnus rugosa var. americana (Regel) Fernald	Alnus incana subsp. rugosa (Du Roi) R.T.Clausen	Alnus rugosa var. americana (Regel) Fernald	*Alnus incana*
*Alnus kolaensis* Orlova	Alnus incana subsp. kolaensis (Orlova) Á.Löve & D.Löve	*Alnus kolaensis* Orlova	*Alnus incana*
Alnus incana f. acuminata (Regel) Regel	Alnus incana subsp. incana	Alnus incana f. acuminata (Regel) Regel	*Alnus incana*
Alnus incana var. virescens S.Watson	Alnus incana subsp. tenuifolia (Nutt.) Breitung	Alnus incana var. virescens S.Watson	*Alnus incana*
*Viola canina* L.	*Viola canina* L.	*Viola canina* L.	*Viola canina*
Viola canina var. montana (L.) Lange	Viola canina subsp. ruppii (All.) Schübl. & Martens	Viola canina var. montana (L.) Lange	*Viola canina*
Viola canina subsp. canina	Viola canina subsp. canina	Viola canina subsp. canina	*Viola canina*
*Viola montana* L.	Viola canina subsp. ruppii (All.) Schübl. & Martens	*Viola montana* L.	*Viola canina*
Viola canina subsp. montana (L.) Hartman	Viola canina subsp. ruppii (All.) Schübl. & Martens	Viola canina subsp. montana (L.) Hartm.	*Viola canina*
*Viola nummulariifolia* All.	*Viola argenteria* Moraldo & G.Forneris	*Viola nummulariifolia* All.	*Viola canina*
*Viola longipes* Nutt.	*Viola adunca* Sm.	*Viola longipes* Nutt.	*Viola canina*
*Salix glauca* L.	*Salix glauca* L.	*Salix glauca* L.	*Salix glauca*
Salix glauca subsp. stipulifera (Flod. ex Hayren) Hiitonen	Salix glauca subsp. stipulifera (Flod. ex Hayren) Hiitonen	Salix glauca subsp. stipulifera (Flod. ex Häyrén) Hiitonen	*Salix glauca*
*Salix pseudolapponum* Seem.	Salix glauca var. villosa Andersson	*Salix pseudolapponum* Seem.	*Salix glauca*
Salix glauca var. acutifolia (Hook.) C.K.Schneid.	Salix glauca var. acutifolia (Hook.) C.K.Schneid.	Salix glauca var. acutifolia (Hook.) C.K.Schneid.	*Salix glauca*
Salix glauca subsp. glauca	Salix glauca subsp. glauca	*Salix glauca* subsp. Glauca	*Salix glauca*
Salix glauca var. cordifolia (Pursh) Dorn	Salix glauca subsp. callicarpaea (Trautv.) Böcher	Salix glauca var. cordifolia (Pursh) Dorn	*Salix glauca*
*Salix labradorica* Rydb.	Salix glauca subsp. callicarpaea (Trautv.) Böcher	*Salix labradorica* Rydb.	*Salix glauca*
Salix glauca subsp. callicarpaea (Trautv.) Böcher	Salix glauca subsp. callicarpaea (Trautv.) Böcher	Salix glauca subsp. callicarpaea (Trautv.) Böcher	*Salix glauca*
*Salix anamesa* Schneid.	Salix glauca subsp. callicarpaea (Trautv.) Böcher	*Salix anamesa* C.K.Schneid.	*Salix glauca*
Salix glauca var. villosa Andersson	Salix glauca var. villosa Andersson	Salix glauca var. villosa (Hook.) Andersson	*Salix glauca*
*Salix glaucops* Anderss.	Salix glauca var. villosa Andersson	*Salix glaucops* Andersson	*Salix glauca*
*Salix desertorum* Richards.	Salix glauca var. villosa Andersson	*Salix desertorum* Richardson	*Salix glauca*
*Salix wyomingensis* Rydb.	Salix glauca var. villosa Andersson	*Salix wyomingensis* Rydb.	*Salix glauca*
Salix glauca var. glauca	Salix glauca var. glauca	Salix glauca var. glauca	*Salix glauca*
Salix glauca var. appendiculata (Vahl) Wahlenb.	Salix glauca subsp. glauca	Salix glauca var. appendiculata (Vahl) Wahlenb.	*Salix glauca*
Salix glauca var. macounii (Rydb.) B.Boivin	Salix glauca subsp. callicarpaea (Trautv.) Böcher	Salix glauca var. macounii (Rydb.) B.Boivin	*Salix glauca*
Salix glauca var. glabrescens (Andersson) C.K.Schneid.	Salix glauca var. villosa Andersson	Salix glauca var. glabrescens (Andersson) C.K.Schneid.	*Salix glauca*
*Salix cordifolia* Banks ex Pursh	Salix glauca subsp. callicarpaea (Trautv.) Böcher	*Salix cordifolia* Banks ex Pursh	*Salix glauca*
Salix glauca var. aliceae C.R.Ball	Salix glauca var. acutifolia (Hook.) C.K.Schneid.	Salix glauca var. aliceae C.R.Ball	*Salix glauca*
Salix glauca var. stenolepis (Flod.) Polunin	Salix glauca subsp. callicarpaea (Trautv.) Böcher	Salix glauca var. stenolepis (Flod.) Polunin	*Salix glauca*
*Salix callicarpaea* Trautv.	Salix glauca subsp. callicarpaea (Trautv.) Böcher	*Salix callicarpaea* Trautv.	*Salix glauca*
Salix glauca subsp. acutifolia (Hook.) Hultén	Salix glauca var. acutifolia (Hook.) C.K.Schneid.	Salix glauca subsp. acutifolia (Hook.) Hultén	*Salix glauca*
Salix glauca var. perstipula Raup	Salix glauca var. acutifolia (Hook.) C.K.Schneid.	Salix glauca var. perstipula Raup	*Salix glauca*
Salix cordifolia var. callicarpaea (Trautv.) Fernald	Salix glauca subsp. callicarpaea (Trautv.) Böcher	Salix cordifolia var. callicarpaea (Trautv.) Fernald	*Salix glauca*
Salix glauca var. stipulata Flod.	Salix glauca subsp. stipulifera (Flod. ex Hayren) Hiitonen	Salix glauca var. stipulata Floderus	*Salix glauca*
Salix glauca subsp. desertorum (Richardson) Hultén	Salix glauca var. villosa Andersson	Salix glauca subsp. desertorum (Richardson) Hultén	*Salix glauca*
Salix glauca var. callicarpaea (Pursh) Dorn	Salix glauca var. callicarpaea (Pursh) Dorn	Salix glauca var. callicarpaea (Pursh) Dorn	*Salix glauca*
*Chenopodium album* L.	*Chenopodium album* L.	*Chenopodium album* L.	*Chenopodium album*
*Chenopodium missouriense* Aellen	Chenopodium album var. missouriense (Aellen) Bassett & Crompton	*Chenopodium missouriense* Aellen	*Chenopodium album*
*Chenopodium lanceolatum* Muhl. ex Willd.	Chenopodium album var. lanceolatum (Muhl.) Coss. & Germ.	*Chenopodium lanceolatum* Muhl. ex Willd.	*Chenopodium album*
Chenopodium album var. album	Chenopodium album var. album	Chenopodium album var. album	*Chenopodium album*
*Chenopodium paganum* Rchb.	*Chenopodium album* L.	*Chenopodium paganum* Rchb.	*Chenopodium album*
Chenopodium album var. reticulatum (Aellen) P.Uotila	*Chenopodium album* L.	Chenopodium album var. reticulatum (Aellen) Uotila	*Chenopodium album*
Chenopodium album var. missouriense (Aellen) Bassett & Crompton	Chenopodium album var. missouriense (Aellen) Bassett & Crompton	Chenopodium album var. missouriense (Aellen) Bassett & Crompton	*Chenopodium album*
*Chenopodium viride* L.	*Chenopodium album* L.	*Chenopodium viride* L.	*Chenopodium album*
Chenopodium album subsp. pedunculare (Bertol.) Arcang.	*Chenopodium album* L.	Chenopodium album subsp. pedunculare (Bertol.) Arcang.	*Chenopodium album*
Chenopodium album var. lanceolatum (Muhl.) Coss. & Germ.	Chenopodium album var. lanceolatum (Muhl.) Coss. & Germ.	Chenopodium album var. lanceolatum (Muhl.) Coss. & Germ.	*Chenopodium album*
Chenopodium album subsp. album	*Chenopodium album* L.	Chenopodium album subsp. album	*Chenopodium album*
*Chenopodium pedunculare* Bertol.	*Chenopodium album* L.	*Chenopodium pedunculare* Bertol.	*Chenopodium album*
*Chenopodium suecicum* Murr.	*Chenopodium suecicum* Murr.	*Chenopodium suecicum* Murr	*Chenopodium album*
*Solanum dulcamara* L.	*Solanum dulcamara* L.	*Solanum dulcamara* L.	*Solanum dulcamara*
Solanum dulcamara var. dulcamara	Solanum dulcamara var. dulcamara	Solanum dulcamara var. dulcamara	*Solanum dulcamara*
*Impatiens capensis* Meerb.	*Impatiens capensis* Meerb.	*Impatiens capensis* Meerb.	*Impatiens capensis*

**Table 2.** List of datasets downloaded from the GBIF for the selected species.

**Dataset key**	**Dataset title**	**Record count**	**Citation**
e45c7d91-81c6-4455-86e3-2965a5739b1f	Vascular Plant Herbarium, Oslo (O), Natural History Museum, University of Oslo	3080	University of Oslo 2021
902c8fe7-8f38-45b0-854e-c324fed36303	Moscow University Herbarium (MW)	1339	Seregin 2021
d29d79fd-2dc4-4ef5-89b8-cdf66994de0d	Vascular plant herbarium TRH, NTNU University Museum	1275	Norwegian University of Science and Technology. 2021
90c853e6-56bd-480b-8e8f-6285c3f8d42b	Field Museum of Natural History (Botany) Seed Plant Collection	1254	Grant and Niezgoda 2020
d415c253-4d61-4459-9d25-4015b9084fb0	The New York Botanical Garden Herbarium (NY)	832	Ramirez et al. 2021
1e61b812-b2ec-43d0-bdbb-8534a761f74c	Canadian Museum of Nature Herbarium	772	Doubt and Torgersen 2021
5c1fdaf6-4a18-4c5d-a84b-a4ba41f077c9	UAM Herbarium (ALA)	619	Ickert-Bond 2021
834c9918-f762-11e1-a439-00145eb45e9a	CSIC-Real Jardín Botánico-Colección de Plantas Vasculares (MA)	179	CSIC-Real Jardín Botánico 2021
95c938a8-f762-11e1-a439-00145eb45e9a	R. L. McGregor Herbarium Vascular Plants Collection	162	Bentley and Morse 2021
07fd0d79-4883-435f-bba1-58fef110cd13	University of British Columbia Herbarium (UBC) - Vascular Plant Collection	160	Jennings 2019
963f12d0-f762-11e1-a439-00145eb45e9a	Botany Division, Yale Peabody Museum	156	Gall 2021
7bd65a7a-f762-11e1-a439-00145eb45e9a	Tropicos Specimen Data	129	Solomon and Stimmel 2021
89c53edb-0fac-4118-bdc0-d70ca50953dc	Kathryn Kalmbach Herbarium	111	Kathryn Kalmbach Herbarium (Denver Botanic Gardens) 2021
4db619a6-9429-4bef-90c9-06cc90c39552	Vascular Plant Herbarium	93	University of Bergen 2021
7e380070-f762-11e1-a439-00145eb45e9a	Natural History Museum (London) Collection Specimens	57	Natural History Museum 2021
b5cdf794-8fa4-4a85-8b26-755d087bf531	The vascular plants collection (P) at the Herbarium of the Muséum national d'Histoire Naturelle (MNHN - Paris)	55	MNHN and Chagnoux 2021a
cc09386c-43a4-4a12-8ae4-d25610645250	University of New Mexico Herbarium	51	University of New Mexico Herbarium (UNM) 2021
0348540a-e644-4496-89d3-c257da9ad776	Marie-Victorin Herbarium (MT) - Plantes vasculaires	36	Brouillet and Sinou 2021
27b4ff4b-29c3-4017-9c48-3750861392f7	University of North Carolina at Chapel Hill Herbarium	27	University of North Carolina at Chapel Hill Herbarium (NCU) 2020
966426ce-f762-11e1-a439-00145eb45e9a	Herbarium Senckenbergianum (FR)	25	Senckenberg. 2021
af1b4db0-c8ce-4a95-b700-8a6a02bed9d6	University of South Florida Herbarium (USF)	25	Franck and Bornhorst 2021
1984c441-b52a-4ced-ba2f-9a2c4fa1898b	Central Michigan University	24	Central Michigan University Herbarium 2020
cd6e21c8-9e8a-493a-8a76-fbf7862069e5	Royal Botanic Gardens, Kew - Herbarium Specimens	24	Royal Botanic Gardens, Kew 2021
821cc27a-e3bb-4bc5-ac34-89ada245069d	NMNH Extant Specimen Records	22	Orrell and Informatics Office 2021
040c5662-da76-4782-a48e-cdea1892d14c	International Barcode of Life project (iBOL)	21	The International Barcode of Life Consortium 2016
7827f68d-c981-4023-bace-288a03434044	Intermountain Herbarium (Vascular plants & algae)	21	Utah State University 2021
2fd02649-fc08-4957-9ac5-2830e072c097	Herbier Louis-Marie (QFA) - Collection de plantes vasculaires	17	Damboise 2020
858d51e0-f762-11e1-a439-00145eb45e9a	The Erysiphales Collection at the Botanische Staatssammlung München	17	Staatliche Naturwissenschaftliche Sammlungen Bayerns. 2021a
3c59bd42-7bfd-421b-8da4-275780390e4c	Desert Botanical Garden Herbarium	16	Desert Botanical Garden Herbarium 2021
8278e7bc-f762-11e1-a439-00145eb45e9a	The Fungal Collection of Helga Große-Brauckmann at the Botanische Staatssammlung München	16	Staatliche Naturwissenschaftliche Sammlungen Bayerns. 2021b
83ae84cf-88e4-4b5c-80b2-271a15a3e0fc	Auckland Museum Botany Collection	15	Cameron and Auckland Museum A M 2021
5733a11d-9286-469c-a9f1-9b21c1e57caa	Estonian Museum of Natural History	10	of Natural History E M and Abarenkov 2021
b89d52a2-861d-4388-adad-c0da3d55fc78	University of Florida Herbarium (FLAS)	10	Perkins 2021
85714c48-f762-11e1-a439-00145eb45e9a	Herbarium Berolinense, Berlin (B)	9	Botanic Garden and Botanical Museum Berlin 2017
65bdd8e3-a27b-4b88-998d-dfb27d528206	Flora of the Korean Peninsula	6	Chang and Kim 2021
858c1c6c-f762-11e1-a439-00145eb45e9a	The Collection of Lichenicolous Fungi at the Botanische Staatssammlung München	4	Staatliche Naturwissenschaftliche Sammlungen Bayerns. 2021c
bf2a4bf0-5f31-11de-b67e-b8a03c50a862	Royal Botanic Garden Edinburgh Herbarium (E)	4	Cubey 2018
5d26c04c-d269-4e1a-9c54-0fc678fae56a	Estonian University of Life Sciences	3	Life Sciences E U O and Abarenkov 2021
646858f7-8620-4124-9405-279539aec76c	Herbarium specimens of Société des Sciences Naturelles et Mathématiques de Cherbourg (CHE)	3	MNHN and Chagnoux 2021b
7ba35058-f762-11e1-a439-00145eb45e9a	The Exsiccatal Series "Triebel, Microfungi exsiccati"	3	Staatliche Naturwissenschaftliche Sammlungen Bayerns. 2021d
a92de2e1-647c-43f2-a8b7-ab1c1a6453dd	University of South Carolina, A. C. Moore Herbarium	3	University of South Carolina 2021
4300f8d5-1ae5-49e5-a101-63894b005868	RB - Rio de Janeiro Botanical Garden Herbarium Collection	2	Forzza et al. 2021
707e1918-0999-4f2f-9ad1-22c0be104861	North Carolina State University Vascular Plant Herbarium	2	North Carolina State University Vascular Plant Herbarium (NCSC) 2020
861e6afe-f762-11e1-a439-00145eb45e9a	Harvard University Herbaria: All Records	2	Kennedy 2021
a1480b53-ae89-4997-ab2a-73b3981ca244	University of Balochistan Herbarium	1	University of Balochistan 2020

**Table 3.** Description of the columns provided in the file Suppl. material 2, which explains the problems that caused us to discard several specimen images.

Column label	Column description
RowID	Each entry in the data file.
ImageID	Unique identity for each digital herbarium specimen (In case of multiple entries, measurements made on different leaves within the same digital herbarium specimen).
Image	If there were no image in the digital herbarium specimen, then the column ‘Image’ was updated as ‘No’ and all other possibilities updated as string ‘NA’.
Number of leaves measured	Contains the number of measured leaves in each digital herbarium specimen and, if not measured, updated as ‘NA’.
Remarks_1	Contains remarks: ‘Juvenile leaves’, ‘Saplings’; all other possibilities updated as ‘NA’. The plant produces juvenile leaves in its earlier years (ordinarily small compared to adult leaves). Sapling is a young tree. We excluded juveniles and saplings to avoid bias in the data.
Remarks_2	Remarks_2 contains the remarks: ‘No leaves’, ‘No measurable leaves’, ‘No measurable leaves tape’ and ‘photograph’. No leaves: When digital herbarium specimen has no leaves (only stem). No measurable leaves: When the digital herbarium specimen has no measurable leaves, for example, only overlapping leaves are not measurable with TraitEx. No measurable leaves tape: When the digital herbarium specimen has no measurable leaves, leaves are covered with tape. Photograph: When the downloaded image is a photograph and not a herbarium specimen, all other possibilities were updated as ‘NA’.
Ruler	If there was no or no appropriate ruler (ruler less than 10 cm and pixelated rulers), then the column ‘Ruler’ was updated as ‘No’ and all other possibilities updated as ‘NA’.
Binomial species name for aggregation	Binomial name for aggregating the scientific names on genus level (Based on the columns 'GBIF Backbone Taxonomy scientific name for GBIF records' and 'GBIF Backbone Taxonomy scientific name for iDigBio records').

**Table 4.** The standard error for leaf area, leaf length, leaf width and leaf perimeter of a single leaf measured on the same herbarium specimen 10 times and repeated the same process for seven different digital herbarium specimens with TraitEx.

**Trait**	**Standard error**
Leaf area	0.039663 cm^2^
Leaf length	0.012555 cm
Leaf width	0.005268 cm
Leaf perimeter	0.035444 cm
